# Risk Factors and Managements of Bile Leakage After Hepatectomy

**DOI:** 10.1007/s00268-015-3156-8

**Published:** 2015-07-10

**Authors:** Kazuhiko Sakamoto, Takao Tamesa, Tokumitu Yukio, Yoshihiro Tokuhisa, Yoshinari Maeda, Masaaki Oka

**Affiliations:** Department of Digestive Surgery and Surgical Oncology, Yamaguchi University Graduate School of Medicine, 1-1-1 Minami-Kogushi, Ube, Yamaguchi 755-8505 Japan

## Abstract

**Background:**

The purpose of this study was to retrospectively determine the risk factors and evaluate the management of bile leakage.

**Methods:**

Three hundred and thirty-four patients who underwent hepatectomy for Child classification grade A liver disease, without biliary reconstruction and laparoscopic procedures, between 2003 and 2013 were included. Risk factors were identified using multivariate analysis.

**Results:**

Bile leakage was observed in 30 (9.0 %) patients. Multivariate analysis demonstrated that type of hepatectomy (segmentectomy 1, medial sectionectomy, anterior sectionectomy, or central bisectionectomy) and operating time was independent risk factors for bile leakage. Among 30 patients with confirmed bile leakage, central type leakage that was in communication with the biliary tree occurred in 23 (76.7 %) patients and peripheral type, which was not in communication with the biliary tree, in 7 (23.3 %) patients. Ten patients were treated with only drainage. Endoscopic or percutaneous transhepatic procedures were performed in 15 cases with central type leakage. Ablation treatment using ethanol or minocycline was mainly performed for peripheral type leakage. Four cases with central type leakage had strictures of the right hepatic duct. Two of them were treated with ablation treatment, portal vein embolization, or fistulojejunostomy. Median duration from diagnosis to end of therapy was 77 days (11–323) in central type and 44 days (6–123) in peripheral type leakage, respectively.

**Conclusions:**

Complex hepatectomy and operating time are independent risk factors for postoperative bile leakage. Biliary exploration should be performed as soon as possible after diagnosis, because most bile leakage is the central type. Central type of bile leakage is sometimes refractory to therapy, needing various treatments and requiring a long time for recovery.

## Introduction

Bile leakage is a complication that is peculiar to hepatectomy. A recent study on a case series of hepatectomies without biliary reconstruction reported that the incidence of bile leakage was between 3.6 and 10 % [1–6]. Bile leakage remains a major cause of postoperative morbidities, such as abdominal abscesses, often leading to a prolonged hospital stay, delayed removal of abdominal drains and seriously affecting patients’ postoperative quality of life. Fujimura et al. described the insertion of a cystic duct tube (C tube), via the cystic duct into the common bile duct, for biliary decompression [7]. Hotta et al. subsequently used the C tube to reduce the incidence of bile leakage in patients who underwent hepatic resection [8]. Therefore, we performed postoperative bile drainage from the cystic duct using a C tube in cases intraoperatively judged to have a high risk for bile leakage. The management of bile leakage has gradually changed from conservative treatment, such as drainage, to interventional strategies, such as endoscopic or percutaneous procedures.

The aims of this study were to retrospectively determine the risk factors and evaluate the management of bile leakage.

## Methods

### Patients

A retrospective analysis of 334 patients with Child classification grade A liver disease undergoing hepatectomy without biliary reconstruction between 2003 and 2013 at the Department of Digestive Surgery and Surgical Oncology, Yamaguchi University Graduate School of Medicine, was performed. All patients were followed up for 90 days to check for bile leakage-associated events. Written informed consent was obtained from all study patients.

### Surgical procedures

Intermittent pedicle clamping (Pringle maneuver) or selective clamping of the pedicles for the segment to be resected was only performed in cases of significant bleeding. Parenchymal transection was performed using a cautery with irrigation forceps (CIF) between 2003 and 2006 [9]. Topical hemostatic agents, such as fibrin sealant glue patches, were applied to the parenchymal cut surface. In recent years, an electrosurgical device (VIO300D; ERBE Elektromedizin, Tübingen, Germany) containing the monopolar soft-coagulation and bipolar clamp coagulation systems has been developed [10, 11]. The VIO system was introduced and used at our hospital between 2007 and 2013. The indications of C tube (6 Fr, 50 cm, Sumius, Sumitomo Bakelite Co., Tokyo, Japan) insertion were hepatectomy requiring cholecystectomy, and cases judged intraoperatively by the surgeon to be at a high risk for bile leakage (The high risk was determined by identification of bile at the cut surface of the liver, an exposed major Glisson’s sheath, or if the cut surface of the liver was broad or complex). After hepatectomy, the cystic duct was cut and the tip of the C tube was inserted into the common bile duct and fixed with an elastic thread. An intraoperative bile leakage test from the C tube was not performed in our series. Basically, a continuous suction device (J-VAC Suction Reservoir; Ethicon, Inc., Johnson and Johnson Company, Somerville, NJ) was placed at the liver transection plane or subphrenic space. However, since various studies have reported that abdominal drainage after hepatectomy is contraindicated [12–16], from 2007 onwards, drains were not placed for the cases judged by the surgeon during the operation as fulfilling the following criteria: one simple cut on the surface of the liver, and postoperative percutaneous puncture being possible. For comparison, the study interval was divided into an early period (2003–2006) and a later period (2007–2013).

### Postoperative management and definition of bile leakage

Abdominal drains were removed from postoperative day (POD) 3 to POD5 if the drain fluid was grossly serous or the total bilirubin level in the drain was less than three times the serum total bilirubin level on POD3, as defined by the International Study Group of Liver Surgery (ISGLS) [17]. Measurement of total bilirubin level in the drain was left to the discretion of the attending surgeons. However, drains were retained in situ if contamination of bile was suspected clinically (Drain fluid was grossly bilious in color or the total bilirubin level in the drain was three times greater than the serum level on or after POD3). For cases with suspected bile leakage, a computed tomography (CT) scan was performed to detect abnormal fluid collection and the drain was exchanged for a new drainage catheter from POD7 to POD10. If the drain exchange failed, or symptomatic fluid collection was confirmed after removal of the drain, or in cases in which a drain had not been placed, CT or ultrasound (US)-guided percutaneous drainage was performed. After diagnosis of bile leakage, if the leaky bile ducts were confirmed to be in communication with the biliary tree by fistulography via the drainage catheter, endoscopic retrograde cholangiography (ERC), or percutaneous transhepatic cholangiography (PTC), the bile leakage was defined as “central type.” If the leaky bile ducts were not in communication with the biliary tree, we defined this bile leakage as “peripheral type.” If the patient’s condition was good after removal of the abdominal drain, the C tube was clamped from POD7 to 10 and then removed after confirming no change in the patient’s condition. Hepatic fibrosis was classified by pathologists as follows: no fibrosis (F0), portal fibrosis without septa (F1), portal fibrosis with few septa (F2), numerous septa without cirrhosis (F3), and cirrhosis (F4) [18].

### Statistical analyses

Continuous data were expressed as median and range values, and were analyzed using the Mann–Whitney *U* test. Categorical data were analyzed using the *χ*^2^ test or Fisher’s exact test. Variables with *p* < 0.05 on univariate analysis that was potentially predictive of bile leakage were then entered into the multivariate logistic regression model. The cut-off value of continuous variables was determined using the receiver operating characteristics (ROC) curves and the optimal cut-off points were determined using the minimum distance from the upper-left corner to any point on the ROC curve. Odds ratio (OR) and 95 % confidence interval (CI) were also calculated. A value of *p* < 0.05 was considered statistically significant. All statistical calculations were performed with the IBM SPSS Statistics Version 22.0 software package (IBM Japan Inc., Tokyo, Japan).

## Results

### Risk factors of bile leakage

Patient characteristics are summarized in Table 1. In all 334 patients studied, the median age was 68 years (range 32–87) and the proportion of male patients was 72.5 % (*n* = 242). The majority of patients had hepatocellular carcinoma (*n* = 245: 73.4 %). Metastatic liver tumor was present in 59 (17.7 %) patients, of whom 50 patients had metastatic colorectal cancer. A hepatic fibrosis grade of F3 or F4 was diagnosed in 149 (44.6 %) patients. The median operating time and blood loss were 350 min (range 76–1028) and 498 g (range 13–9425), respectively. C tubes were used in 157 (47.0 %) patients, with no serious complications related to C tube insertion and removal being observed. Median hospital stay was 23 days (range 10–181). Three patients (0.9 %) died in the hospital from postoperative complications. Of these, one patient died within 30 days. Bile leakage was observed after hepatectomy in 30 (9.0 %) patients. Comparison of perioperative factors between groups with and without bile leakage revealed a significantly increased risk of bile leakage in patients with operating time (*p* < 0.01) and blood loss (*p* = 0.049), respectively. There were no difference in terms of study period (*p* = 0.17) and C tube usage (*p* = 0.14) in patients with and without bile leakage.

**Table 1:** Characteristics of patients with and without bile leakage

	Total population (*n* = 334)	Bile leakage (−) (*n* = 304)	Bile leakage (+) (*n* = 30)	*p* value
Age	68 (32–87)	68 (32–87)	70 (39–81)	0.83
Gender				
Male	242	220 (91.3 %)	22 (8.7 %)	0.91
Female	92	84 (90.9 %)	8 (9.1 %)	
Disease				
HCC	245	222 (90.6 %)	23 (9.4 %)	0.52
Metastatic tumor	59	53 (89.8 %)	6 (10.2 %)	
Others	30	29 (96.7 %)	1 (3.3 %)	
Fibrosis staging				
F 0–2	185	167 (90.3 %)	18 (9.7 %)	0.59
F 3–4	149	137 (91.9 %)	12 (8.1 %)	
Glucose intolerance				
No	239	217 (90.8 %)	22 (9.2 %)	0.82
Yes	95	87 (91.6 %)	8 (8.4 %)	
Previous hepatectomy				
No	296	272 (91.9 %)	24 (8.1 %)	0.11
Yes	38	32 (84.2 %)	6 (15.8 %)	
Preoperative TACE, RFA or PEI				
No	266	242 (91.0 %)	24 (9.0 %)	0.96
Yes	68	62 (91.2 %)	6 (8.8 %)	
Period				
2003–2006	128	120 (93.8 %)	8 (6.3 %)	0.17
2007–2013	206	184 (89.3 %)	22 (10.7 %)	
Operation time (min)	350 (76–1028)	343 (76–1015)	444 (266–1028)	<0.01*
Blood loss (g)	498 (13–9425)	470 (13–4944)	740 (145–9425)	0.049*
Use of C tube				
No	177	165 (93.2 %)	12 (6.8 %)	0.14
Yes	157	139 (88.5 %)	18 (11.5 %)	
Mortality				
No	331	302 (91.2 %)	29 (8.8 %)	0.25
Yes	3	2 (66.7 %)	1 (33.3 %)	

Data are presented as absolute numbers (percentage) or median (range minimum–maximum)

*HCC* hepatocellular carcinoma, *TACE* transcatheter arterial chemoembolization, *RFA* radiofrequency ablation *PEI* percutaneous ethanol injection

* *p* < 0.05

Partial hepatectomy or segmentectomy (2, 3, 5, 6, 7, or 8) was performed in 156 (46.7 %) patients, (extended) left hepatectomy in 42 (12.6 %) patients, posterior sectionectomy or (extended) right hepatectomy in 75 (22.5 %) patients, and segmentectomy 1, medial sectionectomy, and anterior sectionectomy or central bisectionectomy in 61 (18.3 %) patients (Table 2). Patients who underwent segmentectomy 1, medial sectionectomy, anterior sectionectomy, or central bisectionectomy were found to be at a high risk for bile leakage (*p* = 0.015).

**Table 2:** Type of hepatectomy in patients with and without bile leakage

	Bile leakage (−) (*n* = 304)	Bile leakage (+) (*n* = 30)		p value
		Central type (*n* = 23)	Peripheral type (*n* = 7)	
Type of hepatectomy				0.015
Partial hepatectomy	149 (95.5 %)	7 (4.5 %)		
Segmentectomy 2, 3, 5, 6, 7, or 8		5	2	
Left hepatectomy (extended)	37 (88.1 %)	5 (11.9 %)		
		5	0	
Posterior sectionectomy	68 (90.7 %)	7 (9.3 %)		
Right hepatectomy (extended)		3	4	
Segmentectomy 1	50 (82.0 %)	11 (18.0 %)		
Medial sectionectomy		10	1	
Anterior sectionectomy				
Central bisectionectomy				

Data are presented as absolute numbers

**Fig. 1:**
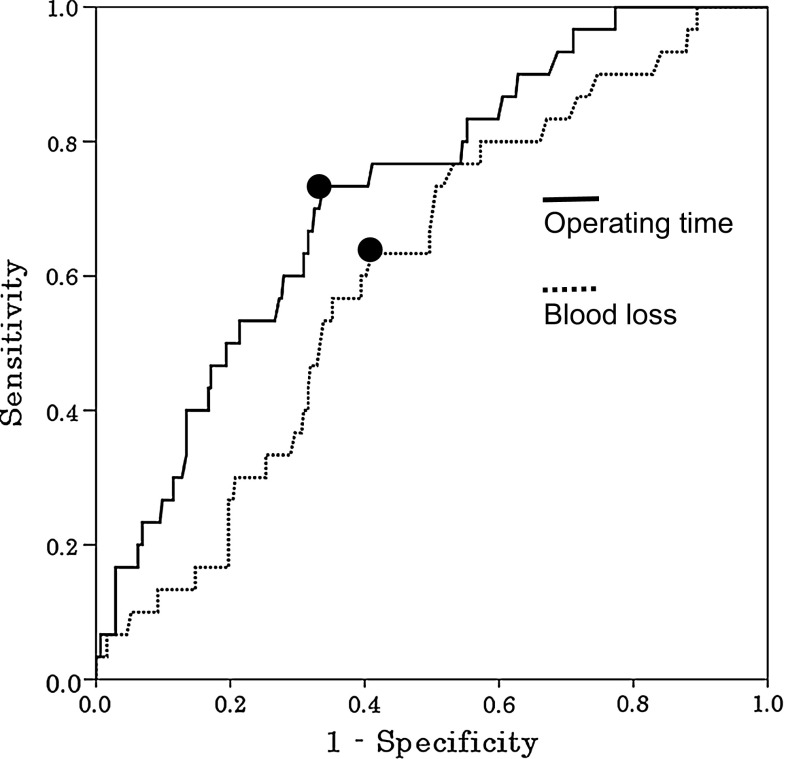
Receiver operating characteristic curve analysis for operating time and blood loss

ROC curve analysis indicated that the optimal cut-offs for operating time and blood loss were 384 min and 628 g, yielding 73.3 % sensitivity and 66.1 % specificity, and 63.3 % sensitivity and 58.9 % specificity, respectively, for the occurrence of bile leakage (Fig. 1). Multivariate analysis demonstrated that type of hepatectomy and operating time (≥384 min) were independent predictors of bile leakage (Table 3).

**Table 3:** Factors predicting the development of bile leakage on multivariate logistic regression analysis

Factor	OR	95 % CI	*p* value
Type of hepatectomy			
Segmentectomy 1, medial sectionectomy, anterior sectionectomy, or central bisectionectomy	2.3	1.0–5.3	0.049*
Operation time (min) (≥384 vs. <384)	4.2	1.6–10.6	0.003*
Blood loss (g) (≥628 vs. <628)	1.4	0.58–3.3	0.47

*OR* odds ratios, *CI* confidence interval

* *p* < 0.05

### Management of bile leakage

**Fig. 2:**
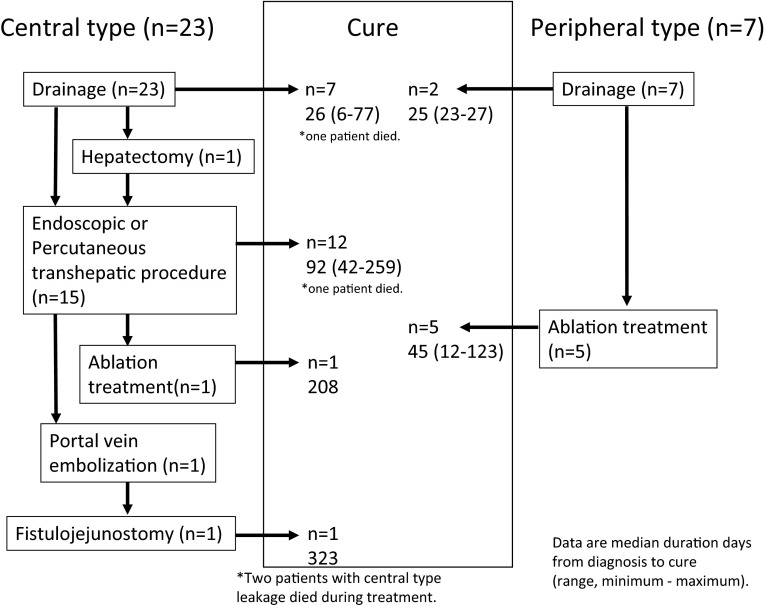
Among patients with central type bile leakage, seven of them were successfully treated by only drainage (one patient died during drainage). After drainage, endoscopic or percutaneous transhepatic procedures were performed in 15 patients, 12 of whom were cured (one patient died during treatment). In two patients who were difficult to cure by this treatment, ablation treatment, portal vein embolization, or fistulojejunostomy were performed. Two patients with peripheral type bile leakage were cured by drainage alone. After drainage, ethanol or minocycline ablation was performed in five patients, the procedure being successful in all of them. “Cure” is defined as the time until drainage tube is completely removed

Among 30 patients with confirmed bile leakage, central and peripheral type leakages were observed in 23 (76.7 %) and 7 (23.3 %) patients, respectively (Table 4). Thirteen (43.3 %) patients were diagnosed with bile leakage from the drain discharge (gross finding: 4 patients, drain/serum total bilirubin ratio: ≥3.0:9 patients). The other 17 (56.7 %) patients were diagnosed by drain exchange (7 patients) or percutaneous drainage after operation (10 patients), respectively. Our treatment process for bile leakage is shown in Fig. 2. The leaky bile ducts were confirmed to be in communication with the biliary tree, indicating central type leakage, in 23 patients. Of them, 7 patients were cured by drainage alone, and reoperation was performed in only 1 patient, in whom; however, primary closure of the leakage site was unsuccessful. In this patient, an endoscopic procedure was performed after reoperation. Biliary drainage (a stent or nasobiliary drain) was performed to decompress the biliary tree in the remaining 15 patients. In 12 of these patients, the bile leakage healed within a median period of 92 days (range 42–259 days). Fistulography revealed that the leaky bile ducts were not in communication with the biliary tree in 7 patients, indicating peripheral type leakage. Two of these patients were cured by drainage alone. Ablation treatment using ethanol or minocycline was performed for the remaining 5 patients, in whom the bile leakage healed within a median of 45 days (range, 12–123 days). In both central and peripheral type bile leakage, fistulography was routinely performed once a week until drain removal, and the drain was clamped when bile leakage had almost disappeared. After a few days, the drain was removed if there was no increase in the inflammatory response.

**Table 4:** Characteristics of bile leakage

	Bile leakage (*n* = 30)	
	Central type (*n* = 23)	Peripheral type (*n* = 7)
Median duration from operation to diagnosis of bile leakage (days)^a^	7 (1–70)	6 (3–24)
Diagnosis		
Gross finding	4	0
Drain/serum total bilirubin ratio ≥ 3.0	3	6
Drain exchange	7	0
Percutaneous drainage	9	1
Median duration from diagnosis to end of therapy (days)^a^	77 (11–323)	44 (6–123)
Treatment^b^		
Only drainage	8 (1)^c^	2
Endoscopic or	15 (1)^c^	0
Percutaneous transhepatic procedure		
Portal vein embolization	1	0
Hepatectomy	1	0
Fistulojejunostomy	1	0
Ablation treatment	2	5

^a^Data are presented as median (range minimum–maximum)

^b^There are duplicated cases

^c^Two patients with central type leakage died during treatment

We experienced four cases with strictures of the right hepatic duct, all of whom had undergone anterior sectionectomy. In these patients, it seemed that the bile duct stump at the cut surface had ruptured due to the increase in internal pressure resulting from the biliary stricture. In two of the four cases, placement of a stent beyond the stricture was successful in several attempts. However, treatment of the remaining two cases failed. In one of the two failed cases, due to gradual decrease in size of the residual posterior segment with the treatment process, ablation of the posterior segment was performed. In this case, the drain was removed 208 days after diagnosis. In the last case, we performed portal vein embolization (PVE) for the posterior segment to eliminate the production of bile [19, 20]. With this, although the volume of bile leakage decreased from 300 ml to 150 ml/day, the leakage persisted. Finally, reoperation was performed, although primary closure of the bile leakage site was impossible because of severe adhesions. Hence, we created an anastomosis between the jejunum and the fistula, using the drainage catheter as a guide. Thereafter, the bile leakage improved and the patient was discharged 323 days after diagnosis.

Central type of bile leakage was most commonly found after segmentectomy 1, medial sectionectomy, anterior sectionectomy, or central bisectionectomy [10 (43.5 %) of 23 patients] (Table 2). Peripheral type of bile leakage tended to occur with right-sided hepatectomy (posterior sectionectomy or right hepatectomy) (4 (57.1 %) patients).

## Discussion

This study focused on the frequency and risk factors of bile leakage after hepatectomy, in patients who underwent open hepatectomy without biliary reconstruction. Previous studies reported that the frequency of bile leakage after hepatectomy ranged from 3.6 to 10 % [1–6] and the rate in this study was within that range. In this study, type of hepatectomy (segmentectomy 1, medial sectionectomy, anterior sectionectomy, or central bisectionectomy) and operation time were independent risk factors for bile leakage.

The correlation between the type of hepatectomy and postoperative bile leakage has not yet been clearly defined. Lo et al. reported left-sided hepatectomy as an independent risk factor for the onset of postoperative bile leakage, because of the risk of damaging the right posterior biliary duct that drains into the left hepatic duct [21]. Hepatectomies in which the cut surface exposes the major Glisson’s sheath and includes the hepatic hilum (central bisectionectomy, anterior sectionectomy, segmentectomy 1, and hepatectomy including segments 4, 5, and 8) are independent risk factors for bile leakage [5, 22]. Hepatectomies including segment 4 usually expose the major Glisson’s sheath and hepatic hilum on the cut surface, with a high risk of damaging the bile duct wall [1]. Left trisectionectomy is a high-risk type of hepatectomy with respect to bile leakage [23]. The present study showed that central type of bile leakage was found after complex hepatectomies, such as segmentectomy 1, medial sectionectomy, anterior sectionectomy, or central bisectionectomy, and those with a broad cut surface, similar to the results reported by others. Hepatectomies that require exposure of the major Glisson’s sheath has the potential to damage the bile duct. It may be useful basic clamp crushing technique in order not to damage the small branches from the hepatic hilum [22]. In this study, refractory bile leakage of a central type, requiring invasive treatment, occurred secondary to latent strictures of the biliary tree. With regard to operative procedures, the Glisson’s sheath may be damaged by devices that generate heat. Hence, such devices should be used carefully when transecting the hepatic parenchyma or attempting hemostasis, especially in the vicinity of the Glisson’s sheath.

There are several reports of ethanol ablation for the treatment of peripheral type of bile leakage, although few reports have mentioned the cause of peripheral type leakage [24–28]. An evaluation of these reports showed that peripheral type of bile leakage mainly developed in right-sided hepatectomy or hepatic resection in which the caudate lobe is cut, which is similar to our results. Hence, peripheral type leakage might potentially have occurred due to damage to the bile duct in the caudate lobe.

Hotta et al. reported that transcystic duct tube drainage after hepatectomy is useful for decreasing postoperative bile leakage [8]. They observed bile leakage in 3.6 % of patients with transcystic duct tube drainage and in 26.3 % of patients without drainage. C tube drainage decompresses intraductal pressure, which leads to prevention of bile leakage from the biliary branches [29, 30]. In this study, there was no difference in the frequency of bile leakage in patients with and without C tube insertion. Nanashima et al. also reported that bile leakage rate was not different between the non-C tube and C tube groups (7.9 vs. 8.4 %) [31]. Further prospective and randomized studies are necessary to clarify the usefulness of C tube insertion.

Previously, drainage was performed for both central and peripheral types of bile leakage. However, since biliary drainage for decompression of the biliary tree for treatment of central type leakage has been reported [32–35], it is recommended that biliary drainage following ERC is performed after diagnosis of bile leakage. Because the timing of ERC or PTC was decided by each attending surgeons, the median duration of treatment using biliary drainage was long (median 92 days). Recently biliary exploration has been performed within one week after diagnosis. Most cases of central type bile leakage were cured by this treatment. However, in cases with refractory strictures of the main bile duct (right hepatic duct), more aggressive and timely therapies (PVE or relaparotomy) should be carefully considered. Ablation treatment should only be performed for peripheral type leakages with no communication with the biliary tree, because ethanol affects the remaining bile duct and causes irreversible damage. Consequently, it is necessary to definitively confirm that the leaking bile ducts are not in communication with the biliary tree by performing several fistulographies or ERC.

Bile contamination can be detected grossly when the total bilirubin level in the drain reaches approximately 10 mg/dl [23]. This finding supports the practice of routine measurement of the total bilirubin level in the drain. Our definition of bile leakage does not strictly follow the ISGLS criteria, because the bilirubin concentration in drainage fluid was not routinely measured. Previous studies, including our results, in which bile leakage was defined by the gross inspection of the drain fluid, may have produced unreliable results. Now that we have a universal definition of bile leakage using objective bilirubin levels of drainage fluid, we should strictly follow it.

In various randomized controlled trials, the overall morbidity was no significant difference between the groups placed and not placed by abdominal drainage, or even higher in the group placed with drainage [12–16]. Considering these reports, abdominal drainage after hepatectomy is unnecessary. We have not placed abdominal drainage for fairly selected cases from 2007. Among 41 patients not placed with drainage, bile leakage was observed in only one patient (2.4 %). However, thirteen (43.3 %) of 30 patients with confirmed bile leakage was revealed by abdominal drainage (gross finding: 4 patients, drain/serum total bilirubin ratio: 9 patients). Dokmak et al. reported that intraoperative bile leakage was risk factor of bile leakage [36]. Although the value of abdominal drainage in patients undergoing hepatectomy remains controversial, its use after procedures with such high risk including factors revealed in this study of bile leakage seems justified.

In conclusion, complex hepatectomy and operating time are independent risk factors for postoperative bile leakage. Biliary exploration should be performed as soon as possible after diagnosis, because most bile leakage is the central type. Central type of bile leakage is sometimes refractory to therapy, needing various treatments and requiring a long time for recovery.
